# Research Progress on Up-Conversion Fluorescence Probe for Detection of Perfluorooctanoic Acid in Water Treatment

**DOI:** 10.3390/polym15030605

**Published:** 2023-01-24

**Authors:** Tan Mao, Xiaoting Shi, Liyuan Lin, Youliang Cheng, Xueke Luo, Changqing Fang

**Affiliations:** 1 College of Mechanical and Precision Instrument Engineering, Xi’an University of Technology, Xi’an 710048, China; 2 College of Mechanical and Material Engineering, North China University of Technology, Beijing 100144, China

**Keywords:** PFOA, fluorescence detection, fluorescent probe, rare earth up-conversion fluorescent materials

## Abstract

Perfluorooctanoic acid (PFOA) is a new type of organic pollutant in wastewater that is persistent, toxic, and accumulates in living organisms. The development of rapid and sensitive analytical methods to detect PFOA in environmental media is of great importance. Fluorescence detection has the advantages of high efficiency and low cost, in which fluorescent probes have excellent fluorescence properties, excellent bio-solubility, and remarkable photostability. It is necessary to review the fluorescence detection routes for PFOA. In addition, the up-conversion of fluorescent materials (UCNPs), as fluorescent materials to prepare fluorescent probes with, has significant advantages and also attracts the attention of researchers, however, reviews related to their application in detecting PFOA and comparing them with other routes are rare. Furthermore, there are many strategies to improve the performance of up-conversion fluorescent probes including SiO_2_ modification and amino modification. These strategies can enhance the detection effect of PFOA. Thus, this work reviews the types of fluorescence detection, the design, and synthesis of UCNPs, their recognition mechanism, properties, and their application progress. Moreover, the development trend and prospects of these detection probes are given.

## 1. Introduction

**Figure 1:**
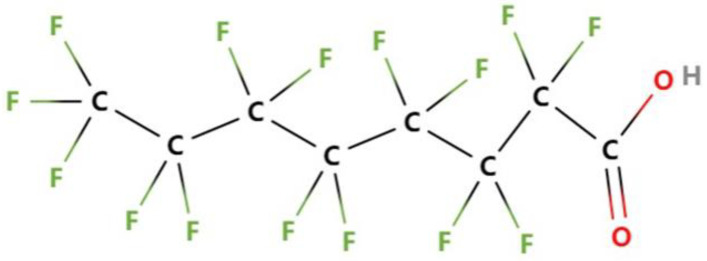
The chemical structure of PFOA.

The chemical formula of PFOA is C_8_HF_15_O_2_ and its structure is shown in Figure 1. PFOA contains 15 F atoms and 8 C atoms. The C-F bond of PFOA has strong polarity and bond energy. Fluorine surfactants of it has a higher surface activity, stronger hydrophobicity, and oleophobicity than traditional surfactants. They are widely used in the chemical, textile, leather, paper making, and cosmetics industries. The fluorinated surfactants of PFOA have the strongest surface activity and the most extensive application. PFOA is hard to degrade because of its strong chemical stability and biological inertness, and can withstand strong light, heat, chemistry, microbial action, and has a high biological metabolism. Therefore, PFOA has bioaccumulation and toxic effect, which will threaten the ecosystem and human health.

PFOA is the main active ingredient of waterproof, oil-proof, and stain-proof finishing agents, which were used in the coatings of various special garments, fabrics, and carpets. Adding PFOA into the coating can improve the wettability, the dispersibility, the uniformity of the color carrier, and prevent caking. The most familiar one is the “Non-stick pan” with a polytetrafluoroethylene (PTFE) surface coating. PFOA is one of the processing aids of PTFE [1]. Due to the applications of PFOA in many fields, PFOA enters the rivers, lakes, and even into drinking water. Li’s group [2] found that PFOA levels in many sewage discharges in Chongqing seriously exceeded the standard and was extremely difficult to deal with. Wang’s group [3] found that the contents of PFOA and PFOS in drinking water in some areas were too high. PFOA is accumulative and transferable, so it can be found in the air, soil, and water [4,5,6,7]. This will increase the possibility of PFOA entering the body.

The damage of PFOA to the human body is enormous. PFOA can enter the human body through the skin surface to cause PFOA to accumulate in the body, which may cause cancer. Sun’s group [8] found that exposure to PFOA leads to the accumulation of ROS in BRL-3A cells, which ultimately leads to the death of the experimental subject. In addition, PFOA has an impact on the human stomach, liver, and nervous system, even leading to cognitive abnormalities [9]. It also affects human sperm [10].

In 2006, the Environmental Protection Agency (EPA) of the United States proposed a reduction plan for PFOA [11]. In 2014, the Norwegian Environmental Protection Agency issued a national ban on the use of PFOA products. In 2016, the United States Toxic Substances Control Act (USTSCA) included PFOA on the list of toxic chemicals [12]. In 2015, China generated large quantities of fluoropolymers, and the consumption of PFOA reached 200 tons [13,14]. Frederick Pontius [15] analyzed in detail the requirements of PFOA and PFOS of various international organizations in recent years, and the regulatory agencies of more than 12 countries formulated the consultation of PFOA in drinking water or groundwater. PFOA was included on the third drinking water contaminant candidate list (CCL). US-EPA restricted the content of PFOA in healthy drinking water at 70 ng/L. PFOA has attracted worldwide attention since 2016. The requirements for PFOA content in drinking water in China are still improving [16,17,18].

The detection of PFOA has a great social significance to people’s lives, but the effectiveness of the detection methods is different. Therefore, the simplest, quickest, and most sensitive methods need to be explored [19,20].

## 2. Method of Detecting PFOA

Traditional methods of detecting PFOA include chromatography, mass spectrometry, and chromatography–mass spectrometry. These methods often need to use expensive instruments to detect due to the unique properties of PFOA [21,22,23,24,25]. The fluorescence detection method is a new way to detect trace elements, and PFOA as a trace element can be detected by it.

### 2.1. Liquid Chromatography-Mass Spectrometry

#### 2.1.1. High Performance Liquid Chromatography–Tandem Mass Spectrometry (HPLC–MS/MS)

**Figure 2:**
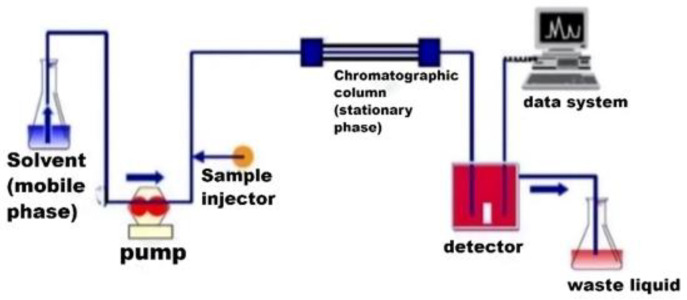
Detection flow chart of HPLC.

High performance liquid chromatography (HPLC) has the advantages of high accuracy, a wide separation range, and low destruction to the material structure. Figure 2 shows the detection flow chart of HPLC. The sensitivity of mass spectrometry (MS) is better than any other analytical method, it is more accurate in characterizing the structure of unknown compounds. Mass separation–mass spectra Characterization (MS–MS) allows further cleavage of the parent atom to obtain information on the cleavage process and molecular structure, often called as tandem mass spectrometry. HPLC–MS/MS combines the advantages of both. The chromatography can be used as a sampling system for the mass spectrometry, and the mass spectrometry as an identifier for the chromatography. HPLC–MS/MS has certain advantages in terms of selectivity and sensitivity [26,27].

#### 2.1.2. High Performance Liquid Chromatography–Quadrupole-Time of Flight-Mass Spectrometry (HPLC–Q-TOF-MS)

The mass analyzer used for HPLC–MS/MS is a low-resolution mass spectrometer, it cannot effectively distinguish the interference when analyzing complex samples. Time of flight-mass spectrometry (TOF/MS) has certain advantages in terms of mass range and resolution, and it has a high mass accuracy and a fast analysis speed. Unlike low-resolution mass spectrometry, TOF/MS greatly improves the anti-interference ability in complex backgrounds and makes the detection results more accurate and reliable [28]. Therefore, HPLCQ–TOF-MS has a certain optimization to analyze samples when in a complex environment, increasing the correctness of detection [29,30].

In 2009, USEPA issued Method 537 to analyze 14 PFAS in drinking water. This method needs to use solid-phase extraction and liquid chromatography/tandem mass spectrometry (LC/MS/MS) [15]. However, this method needs skilled analysts and expensive instruments to get effective results.

### 2.2. Gas Chromatography–Mass Spectrometry

#### 2.2.1. GC-MS

Because the partition coefficient of the sample in the chromatographic is different when it is in the gas or solid phase state. Therefore, GC–MS uses this principle to complete detection by multiple distributions of the sample. In GC–MS, the gas chromatography is used as the sampling system of mass spectrometry, while the mass spectrometer is used for gas chromatography detection. However, the GC–MS detection process is more cumbersome and therefore less used in applied methods [31].

#### 2.2.2. Pre-Column Derivatization–Gas Chromatography

Pre-column Derivatization–Gas chromatography is an improvement on the shortcomings of GC–MS. The derivatization technology can reduce the temperature of the target analyte and improve the signal required to detect the target, which reduces the cost of the detection instrument and can effectively detect PFOA [32], such as silanization, esterification, and acylation [33,34].

### 2.3. Fluorescence Detection

Traditional methods of detecting PFOA have certain advantages, but still have the shortcomings of high cost and low universality. Fluorescence detection is the latest in current detection methods, which can detect target substances conveniently and quickly. Zheng’s group [35] sensitively detected perfluorinated pollutants through fluorescence detection.

The fluorescent probe uses fluorescent substances as indicators, and under the excitation of a certain wavelength of light, the indicator produces fluorescence. Then, the change of fluorescent strength is detected to achieve a qualitative or quantitative analysis of the detected substances. The signal emitted by the reporter fluorophore is absorbed by the quenching fluorophore when the probe is intact, but the two are separated when the probe detects the substance. In other words, the luminescent substance at the top of the fluorescent probe will specifically bind to the detection target when the fluorescent probe reacts with the detection substance, which can cause a fluorescence quenching reaction.

**Figure 3:**
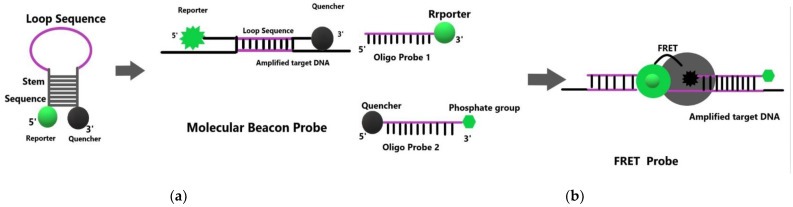
(**a**) Detection principle of Beacon probe (**b**) Detection principle of FRET probe.

The probe can be divided into a Beacon probe and a FRET (fluorescence resonance energy transfer) probe. Beacon mode depends on the molecular beacon accumulating fluorescence in the presence of enzymatic digestion and cannot show the detection status in time, as shown in Figure 3a. However, the detection signal of the FRET mode is a real-time signal, which can display the detection status in time, as in Figure 3b. Therefore, the FRET mode will be selected when detecting trace elements. The FRET probe includes fluorescein probes, inorganic ion fluorescent probes, fluorescent quantum dots, and molecular beacons [36].

Fluorescence quenching can lower the intensity of emitted light from fluorescent molecules. Fluorescence quenching can cause some changes including fluorescence intensity, related excitation peak changes, and fluorescence peak position changes. The substances react with fluorescent materials causing these changes and these processes are called fluorescence quenchers. For example, electron acceptors are fluorescence quenchers in photosynthesis.

Fluorescence quenching can be divided into dynamic quenching and static quenching. Dynamic quenching occurs when the fluorescence quenching between the excited state fluorescent molecule and the quencher will cause an energy transfer or physical collision. However, for the excited state molecule, it is not necessary to complete the direct contact between the two acting molecules. They can create optical collision effects directly. Static quenching refers to the formation of a compound between the ground state fluorescent molecule and the quencher through weak binding, and the compound will completely quench the fluorescence.

**Figure 4:**
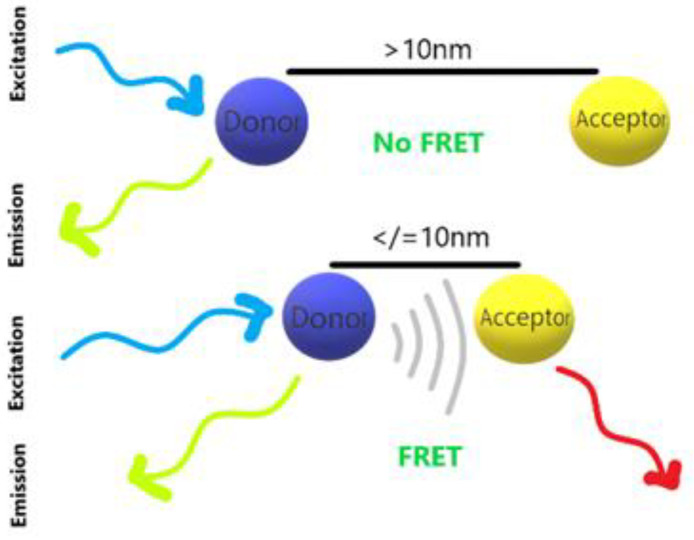
Fluorescence resonance energy transfer (FRET).

The principle of fluorescence quenching is the result of fluorescence resonance energy transfer (FRET) [37,38,39]. As shown in Figure 4, FRET is a non-radiative process formed by the interaction between dipoles and dipoles, and the energy will be generated when donor and acceptor close to each other.

A small molecule fluorescent probe is widely used to detect trace elements. It has the advantages of low cost, simplicity of handling, and a high detection sensitivity. A fluorescent probe made of fluorescent materials has the advantages of good hydrophilicity and multifunctional integration. It plays a significant role in detecting trace elements. A detailed comparison can be seen in Table 1.

Fluorescent sensors use materials that have a fluorescence effect to label the detectors and detect substances through changes in the fluorescence signal. Fluorescent sensors have the advantages of high sensitivity and specificity. Kelsey L. Rodriguez’s group [46] analyzed in detail the various drawbacks of the conventional detection of PFASs such as GS–MS and so on, and analyzed the ability of various sensors to detect PFAs. Table 2 is a summary of the sensors involved in it. The advantages and disadvantages of HPLC–MS/MS, HPLC–Q-TOF-MS, GC–MS, and Pre-column Derivatization GC are also compared in Table 2.

After comparing the detection methods, it was finally found that the fluorescence detection method was the most convenient and efficient, and the fluorescence probe method was more suitable to detect PFOA in wastewater.

As can be seen from Table 2, the detection effect of HPLC–MS/MS, HPLC–Q-TOF-MS, and GC–MS are not ideal and the cost is high. The detection effect of fluorescent sensors and other methods is good, in which the optical sensor is simple to operate, but the detection performance of rare earth up-conversion fluorescent probes is better.

## 3. Fluorescent Materials

### 3.1. Fluorescent Polymer Materials

Fluorescent polymers are prepared by bringing small molecular fluorescent compounds into the side chains and segments of polymers, or using the polymerization of fluorescent functional monomers. Since 1960, the research on fluorescent polymers has included materials science, medicine, and chemistry.

Fluorescent polymers can be used as conversion materials to convert chemical signals into visible electrical or optical signals, making it easier to detect trace elements. In the detection of organic pollutants or small organic molecules, fluorescent polymers can detect trace polymers by FRET. Chen’s [62] group established a two-in-one platform based on conjugated polymer, which can effectively detect perfluoroalkyl substances. The polymer has the advantages of easy preparation and modification. The sensor made of fluorescent polymer has a superior sensing ability. For example, Vinh Van Tran [63] launched a conjugated polymer-based biosensor to solve the challenge of rapid virus detection based on COVID-19, but there are shortcomings. Akhtar Hussain Malik’s group [64] prepared conjugated polymer nanoparticles that can detect nitro-explosive picric acid on multiple platforms. Chen’s group [65] reviewed smart conjugated polymers for optical detection. However, there are shortcomings in the separation and analysis method.

Fluorescent polymers can be divided into water-insoluble fluorescent polymers, water-soluble fluorescent polymers, and amphiphilic fluorescent polymers. Water-insoluble fluorescent polymers have properties of high strength, easy film formation, solvent resistance, heat resistance, and easy processing. In the fluorescence detection of molecules or atoms which need to be performed in a biological environment, water-soluble fluorescent polymers show advantages in this respect [66,67]. The amphiphilic fluorescent polymers have the characteristic that the lipophilic part is incompatible with the hydrophilic part in the structure, which makes microphase separation easy. In addition, amphiphilic fluorescent polymers have fixed fluorescence emissions [68,69].

### 3.2. Rare Earth Up-Conversion Nanomaterials

Rare earth up-conversion nanomaterials (UCNPs) have rich 4f energy levels, in which electrons can transition, so they have rich optical properties and are widely used in optical, electrical, and magnetic fields [70,71]. Up-conversion luminescence is the absorption of low-energy (long-wavelength) photons and the emission of high-energy (short-wavelength) photons, which can also be called a phenomenon of photoluminescence. The famous Stokes theorem shows that materials can only be excited by high-energy light (short wavelength) and emit low-energy light (long wavelength). However, up-conversion luminescence reverses the knowledge of Stokes theorem, so it is called anti-Stokes luminescence [72].

In the mid-1960s, Auzel’s group [73,74] discovered up-conversion luminescence. The study found that adding activators and sensitizers to up-conversion materials could greatly improve the luminescence efficiency of UCNPs. The fluorescent probe prepared by UCNPs has better luminescence effect and stable chemical properties [75,76], and does not undergo photochemical changes resulting in photobleaching. Therefore, the up-conversion fluorescent probe is a new way to study.

#### 3.2.1. Up-Conversion Fluorescence

The luminescence principle of UCNPs can be divided into four situations: the excited state absorption up-conversion process, the energy transfer up-conversion process, the cooperative up-conversion process, and the photon avalanche up-conversion process.

**Figure 5:**
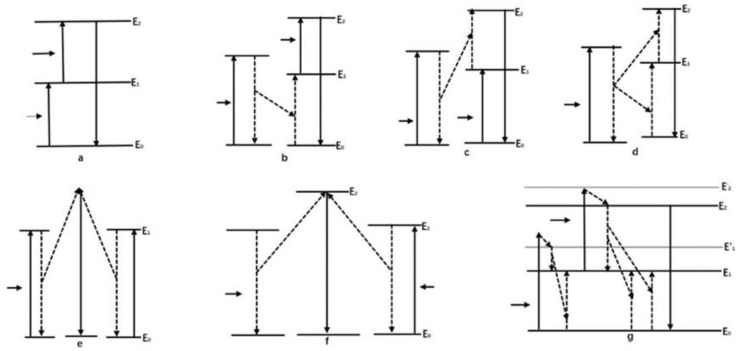
Principle diagram of up-conversion fluorescence. (**a**) The excited state absorption up-conversion process. (**b**) The energy transfer up-conversion process. (**c**) The relaxation up-conversion process. (**d**) The continuous energy transfer up-conversion process. (**e**) The cooperative luminescence up-conversion process. (**f**) The cooperative sensitization up-conversion process. (**g**) The photon avalanche up-conversion process.

The excited state absorption up-conversion process refers to how an ion in the ground state E_0_ absorbs a low-energy photon and is excited to a metastable E_1_. Then, it absorbs a low-energy photon again and is excited to a higher excited state E_2_. Finally, it transitions from E_2_ to E_0_ and radiates high-energy photons. (Figure 5a). In other words, the excited state absorption process is a continuous multiphoton absorption process of a single ion. The same rare earth ion transits from E_0_ to E_2_ through absorbing two-photons or multi-photon absorption, and then releases the energy back to E_0_ in the form of light radiation [77]. The excited state absorption up-conversion process is the most basic process of up-conversion fluorescence. However, the process requires a small concentration of doped atoms to reduce the energy loss of energy transfer between atoms.

The energy transfer up-conversion process uses the excitation light to excite the sensitizer atoms, prompting them to transition from the ground state to the excited state. The electrons in the excited state have a transition down to the lower-energy excited state or back to the ground state, transferring the released energy to the activated ion in the form of a non-radiative FRET, which excites the activated ion to a higher energy state and finally transitions to the ground state producing a radiative transition. Therefore, this process is in line with the luminescence principle of up-conversion fluorescent probes. The energy transfer up-conversion process can be divided into the energy transfer up-conversion process (Figure 5b), the relaxation up-conversion process (Figure 5c), and the continuous energy transfer up-conversion process (Figure 5d). The process requires that the concentration of rare earth atoms doped is high enough to ensure the effectiveness of energy transfer.

The cooperative up-conversion process can get to the high-energy state directly, and then radiate short-wavelength photons. It can be divided into the cooperative luminescence up-conversion process (Figure 5e) and the cooperative sensitization up-conversion process (Figure 5f). The photon avalanche up-conversion process is a process of using ground state and excited state absorption to rapidly increase the number of intermediate sub-stable atoms. Then, the activator achieves a high-energy state particle number layout, and finally has a transition to the ground state radiation photon process, as shown in Figure 5g [78].

UCNPs are mainly composed of activator, sensitizer, and substrate. UCNPs have the advantages of strong photostability, low toxicity, and no damage to biological tissues during use. They can accurately, stably, and safely detect target substances [79].

#### 3.2.2. Composition of UCNPs

(1) substrate

In the energy level transition of the up-conversion luminescence, the substrate material mainly provides the appropriate reaction environment for the activator and sensitizer. The up-conversion luminescence intensity will be improved accordingly when using the substrate material which has better properties because rare earth atoms with 4f level spacing are smaller than other elements. Therefore, the phonon energy of the substrate material must be lower, therefore fluoride and oxide are commonly used. Up-conversion luminescence has a large anti-Stokes displacement. The material needs an intermediate or metastable energy level, which has a long lifetime. At the same time, it also needs a strong excitation light source to realize up-conversion luminescence. The transition between energy levels of rare earth ions to F-F forbidden resistance makes the metastable energy levels of the luminescence center have a long energy level lifetime. This can realize the two-photon or multi-photon effect. In other words, up-conversion materials need more metastable energy levels and stronger excitation light sources for luminescence. The substrate of up-conversion luminescent materials is relatively special, which can reduce the phonon energy. Finally, it can form a large number of metastable energy levels to improve the fluorescence intensity of up-conversion luminescent materials. Therefore, fluoride is usually chosen as the substrate material [80]. NaYF_4_ is one of the highest luminescence efficiency substrates used as up-conversion nanomaterials [81]. NaGdF_4_ not only meets the luminescence efficiency and has paramagnetic properties, but also can be used as a multifunctional contrast agent [82,83].

(2) activator and sensitizer

The activator with less content in UCNPs can make the basic non-luminescent material emit strong light, and can also change the luminescent color and luminescent efficiency of UCNPs. The selection of activators should meet the conditions of more energy level transitions, the full use of infrared light, and the energy exchange with other atoms. The most commonly used activators are Er^3+^, Tm^3+^, and Ho^3+^. Their unique energy level structure makes UCNPs emit emission wavelength, while the sensitize usually uses Yb^3+^. Yb^3+^ can enhance the absorption intensity of rare earth atoms to excited light due to its simple energy level structure, and it can enhance the absorption intensity of rare earth atoms for excitation light. The sensitizer absorbs the energy of photons and transfers the energy to the activator [84,85].

**Figure 6:**
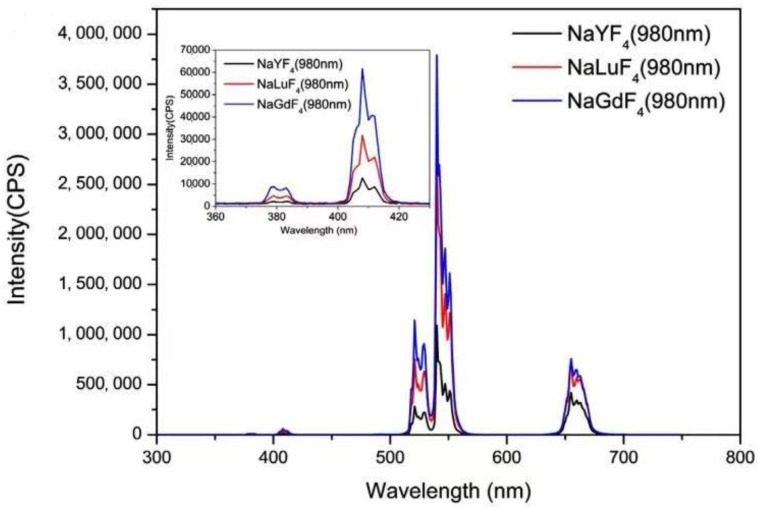
Fluorescence peak of different substrates. Adapted with permission from Nie J et al. [86].

Nie’s group [86] used first principles to study and calculate the luminescence rate of UCNPs prepared by different fluoride substrate materials and found that β-NaGdF_4_ has the best luminescence rate among the three substrates by comparing β-NaYF_4_, β-NaGdF_4_, and β-NaLuF_4_, as shown in Figure 6. Fan’s group [87] carried out further research on β-NaGdF_4_. It was doped with three kinds of systems to make three types of photonic crystals, and their structural properties and optical properties were simulated and compared. They finally found that the optimal preparation was β-NaGdF_4_: Er^3+^/Yb^3+^. Therefore, the luminescent properties of UCNPs obtained from different substrates and activators are also different.

PFOA has rich F atoms, which can make the up-conversion fluorescent probe occur fluorescence quenching by F-F interaction, so as to achieve the purpose of detection. In the detection of Perfluorochemicals, it is found that Ho^3+^ can better match Yb^3+^ to enhance the luminescence intensity of UCNPs [88,89,90].

**Figure 7:**
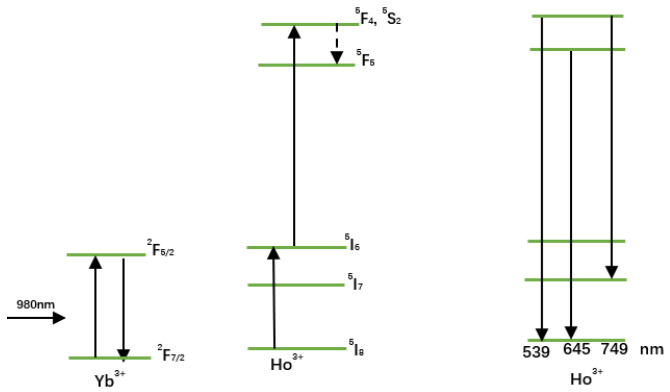
Energy level diagram and energy transfer mechanism of Yb^3+^ and Ho^3+^.

Under 980 nm excitation, the probe produces three emission peaks, in which 541 nm emission from the ^5^S_2_-^5^I_8_ transition of Ho^3+^, 649 nm emission from the ^5^F_5_-^5^I_8_ transition of Ho^3+^, and 750 nm emission from the ^5^S_2_-^5^I_7_ transition of Ho^3+^, as shown in Figure 7. The emission yield of rare-earth up-conversion fluorescent probes can reach 90% and fluorescence quantum yields can reach 5.6%, while core-shell fluorescent probes can reach 95%. The emission yield of rare-earth up-conversion fluorescent probes can be enhanced using functionalized modifications. Table 3 shows a comparison of the fluorescence quantum yields of the different fluorescent probes.

Comparing several fluorescent materials, UCNPs are found to have the best luminescence and do not cause secondary pollution to the environment during the detection process.

## 4. Preparation and Optimization of UCNPs

### 4.1. Method of Preparing UCNPs

(1) Hydrothermal method

**Figure 8:**
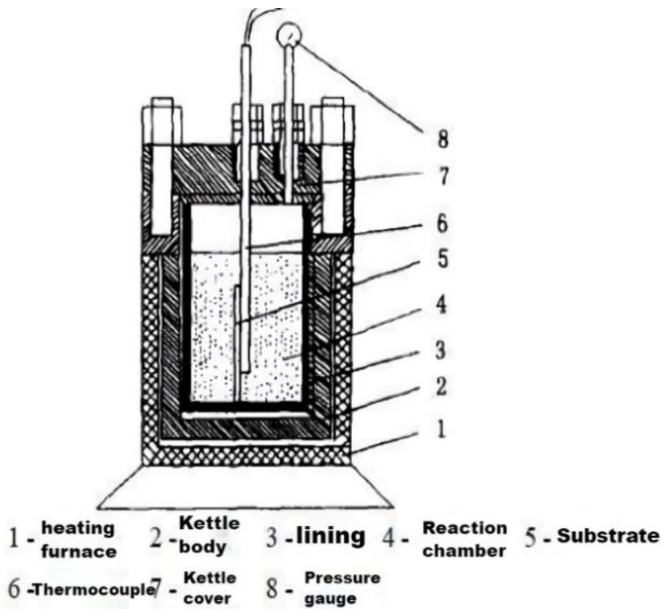
Hydrothermal reactor.

The hydrothermal method can be used in a special sealed high-pressure vessel, such as a vacuum reactor, as shown in Figure 8. This method makes compounds that are difficult to dissolve or cannot be dissolved, and finally generates synthetic nanomaterials with water as the reaction system under high temperature and high pressure. The preparation process of the hydrothermal method is easy to control. The crystallized grains are good and the size of the nanoparticles is uniform [97,98]. Zhang’s group [99] proposed a general strategy for synthesizing nanocrystals in hydrothermal processes using liquid–solid–solution phase transfer and separation, which can also be considered in the preparation process. However, the reaction time of hydrothermal method is too long. It can be slightly improved by increasing the ion concentration correspondingly [100,101].

(2) Solvothermal method

**Figure 9:**
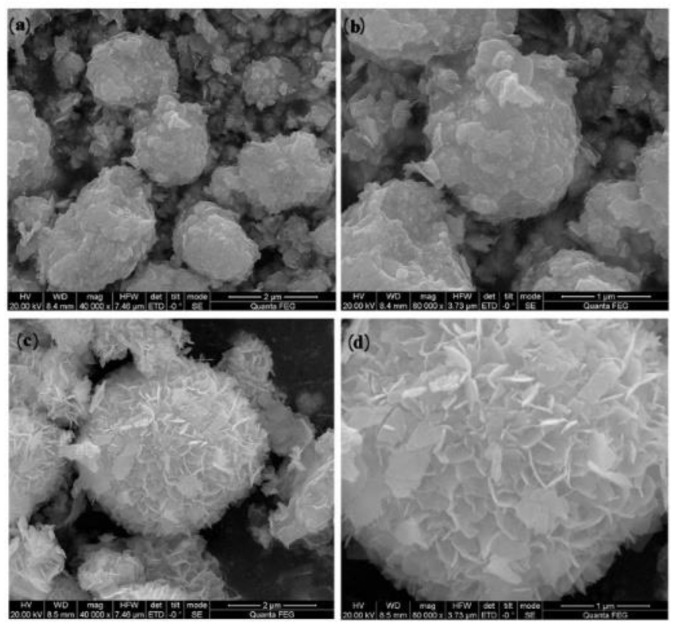
SEM images of Bi_2_O_3_ (**a**,**b**); 1:1-Bi/BiOBr (**c**,**d**). Adapted with permission from Yuan XY et al. [106].

The solvent method is one of the most commonly used methods to prepare UCNPs, which can be used to synthesize lanthanide tetrafluoride UCNPs, such as NaYF_4_, NaGdF_4_, and NaLuF_4_ [102,103,104]. The solvothermal method is similar to the hydrothermal method, but the difference lies in their precursor solution. The solvothermal method uses non-aqueous organic solvents, which can greatly improve the activity of reactants under the set pressure. UCNPs prepared by it have good crystallization, good particle dispersion, dispersion, and small particle size [105]. Figure 9 is an SEM image of Bi_2_O_3_ prepared by the solvothermal method [106,107]. It can be shown that the crystallinity of nanoparticles produced by the solvothermal method is good, but this method has some experimental risks.

(3) Coprecipitation method

**Figure 10:**
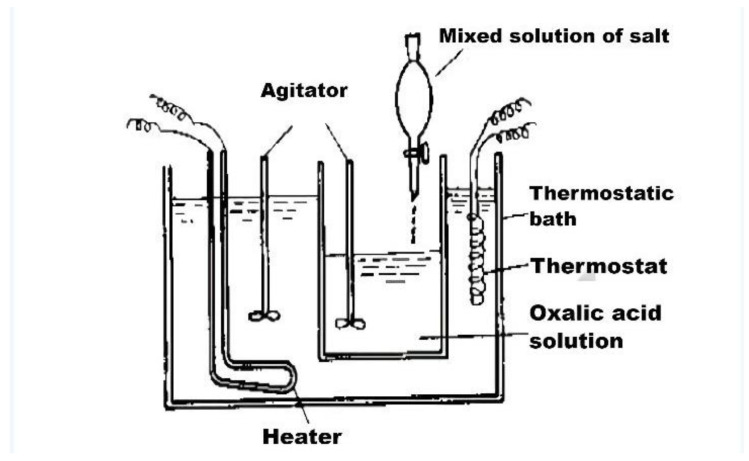
Diagram of compound precipitation synthesis device.

The coprecipitation method refers to the adding of the corresponding precipitant into the solution containing two or more cations and finally producing the target substance through a precipitation reaction. Figure 10 shows the coprecipitation device. NaF, NH_4_F, and other precipitating agents can be added to the preparation of up-conversion nanoparticles. Because the α-phase up-conversion nanomaterials have low luminous efficiency, they need to be calcined and annealed to become β-phase up-conversion nanomaterials which is a way to obtain high-quality nanocrystals [108]. At the same time, sealing ligands such as PVPk30 need to be added to improve their stability [109,110]. The equipment required by this method is easy to obtain and easy to operate, but it has the disadvantage of low crystallinity synthetic and agglomerate [111,112].

(4) Sol–gel method

**Figure 11:**
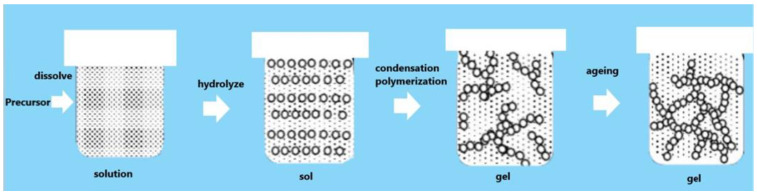
Flow chart of sol-gel method.

The sol–gel method involves the use of chemicals to prepare the precursors of metal water–soluble inorganic salts. It will produce a uniformly distributed liquid when the precursors are placed in a solvent, and the dispersion liquid can be reasonably added to it. An appropriate amount of coagulant can be added to make the salt hydrolyze produce a coagulation process and generate a uniform organic sol. Finally, the uniform organic sol is dried to obtain the application of UCNPs, as can be seen in Figure 11. However, it is difficult to control the morphology and particle size of the final product when applying this method. The method has the advantages of simple operation and low cost, but it has the disadvantages of poor dispersion, long preparation cycles, and the need for specific aging. Even so, the method has the possibility of agglomeration [113].

(5) Microemulsion method

**Figure 12:**
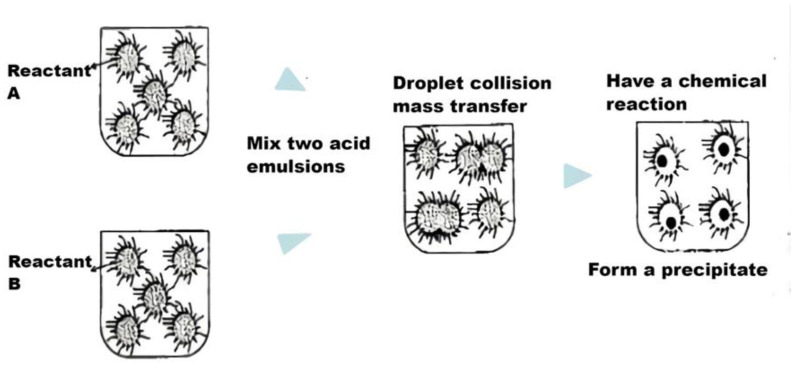
Flow chart of microemulsion method.

The microemulsion method can obtain a new reaction substance by adding certain emulsifiers to two immiscible liquids, which makes the aqueous phase effectively dispersed in the oil phase through the reaction. UCNPs prepared by this method have controllable particle size and a good appearance. For example, Zhou’s group [114] used this method to prepare the nano-SiO_2_ with a narrow particle size distribution and good dispersion. However, the operation is complex and costly, and the required reaction conditions are harsh. The flow chart is shown in Figure 12.

Among the five methods, the hydrothermal method and solvothermal method are simple and fast, which will be used in the practical application of the preparation of UCNPs in the future. Table 4 shows a comparison of the advantages of the common methods used to prepare UCNPs.

### 4.2. Optimization of Luminescence Efficiency of Up-Conversion Nanomaterials

There are three ways to optimize the up-conversion luminescence efficiency. The first way is to modify the surface of up-conversion nanoparticles. Using the atom resonance effect enhances the probability of radiative transition between the energy levels of rare earth atoms.

The second way is to dope some other atoms in the substrate [116,117], such as Gd^3+^ and Mn^3+^ [118]. The third way is to improve the luminous efficiency of materials by using the core-shell cladding and this way is more commonly used. The up-conversion luminescence nanocrystalline is coated with a similar lattice structure of passivated layer to form a large nanocrystalline. In this way, the activator can be effectively confined to the center of the nanocrystalline. This limits its aimless migration to cause quenching. The core-shell structure also greatly modifies the surface defects of nanocrystals, improving the luminescence optimization of up-conversion [119,120].

### 4.3. Surface Functionalization of Up-Conversion Nanomaterials

(1) Hydrophilic modification

Up-conversion nanomaterials are oil-based, and their surfaces are often coated with hydrophobic ligands (OA or OM), making them difficult to be directly used for the detection of pollutants in water treatment. Combined with the water-insoluble property of PFOA, the detection of PFOA is more difficult. The hydrophilic modification of UCNPs can greatly improve their practical application performance. The hydrophilic modification methods include ligand exchange, surface silanization, ligand oxidation, and layer by layer assembly.

Surface silanization can prevent quenching by blocking the contact between nanoparticles and peripheral water, and can make the active groups carry out the next biomolecular coupling. For example, SiO_2_ modification can make the material multi-pore size [121,122]. Ligand oxidation can couple UCNPs to biomolecules without interfering with the morphology and luminescence properties of UCNPs. However, this method is only applicable to the surface of nanoparticles without oxidation, and the modification time is long. In addition, it is easy to aggregate after modification [123]. The ligand removal method is simple and portable, and has little effect on the particle size of UCNPs. UCNPs are easy to aggregate after OA removal [124]. The ligand exchange method can greatly improve hydrophilicity and biocompatibility, but UCNPs will lose part of the luminous intensity after the exchange [125]. The surface-coated amphiphilic polymer method makes UCNPs have a good stability in an aqueous solution, but it will increase the diameter of UCNPs. The method is also difficult and costly [126].

(2) Modification of functionalization

UCNPs should be further functionalized to enhance practical performance when after hydrophilic modification. For example, it can be modified by amino group [76] or carboxyl group modification [127]. These ways can enhance the ability of detection.

### 4.4. Principle of Up-Conversion Fluorescent Probe Detecting of PFOA

**Figure 13:**
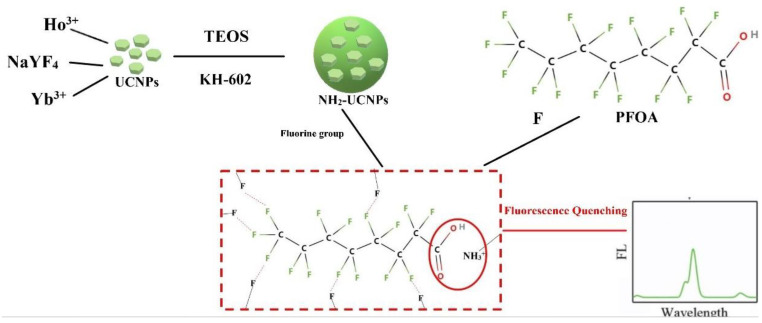
Schematic diagram of detecting PFOA.

PFOA has rich F atoms, and F atoms have a huge electronegativity which makes the carbon-fluorine bond highly polar. Therefore, we can use this characteristic to detect PFOA. If the substrate of the UCNPs is fluoride, the up-conversion fluorescent probe prepared by it has fluorine groups on the surface, as shown in Figure 13. In addition, the amino modification of UCNPs can enhance luminescence. PFOA can bind to up-conversion fluorescent probes through F-F and electrostatic interactions, resulting in fluorescence quenching. Then, the content of PFOA in the aqueous solution can be known by comparing the fluorescence intensity of this process.

Li’s group [96] prepared a mesoporous structure-based up-conversion molecularly imprinted fluorescent probe (NH_2_–UCNPs @MIPs) to detect PFOS. The chemical structure of PFOS is similar to PFOA. They both have 15 F atoms. The difference is that PFOS contains SO_3_^−^ and PFOA contains OH^−^.

PFOS can combine with NH_2_–UCNPs @MIPs using the interaction of F-F and electricity, resulting in its fluorescence quenching. Based on this, they establish an efficient identification and highly sensitive detection technology to detect PFOS which a concentration range of PFOS is 0.01~15 nmol/L. This principle also applies to PFOA detection, because they have similar chemical structures and properties.

## 5. Application Progress and Recovery

### 5.1. Applications of Up-Conversion Fluorescent Probes in Detection

In the aspects of sewage treatment, food safety, and blood detection, detection technology is constantly being improved and innovated, such as through colorimetric analysis [128], enzyme inhibition [129], biochip technology [130], and UCL technology. Up-conversion fluorescent probes have excellent applications in detection.

In sewage treatment, it is necessary to face the detection of multiple sources of pollution, such as pesticides and veterinary drugs which easily flow into the water during use. It will cause clean water to be polluted into dirty water. Wang’s group [131] found that Cu^3+^ can generate a fluorescence quenching reaction with UCNPs, so as to develop a highly sensitive fluorescence probe. Liu’s group [132] found that UCNPs and gold nanoparticles can generate resonance energy transfer to detect cyanogen in sewage. Au nanoparticles will be adsorbed on the surface of UCNPs through electrostatic reaction, which reduces the fluorescence intensity of UCNPs. When up-conversion fluorescent probe detects an aqueous solution containing cyanide, the fluorescence intensity of UCNPs is strengthened. The content of cyanogen is displayed according to the comparison of fluorescence intensity. Up-conversion fluorescent probes have a great role in promoting the field of sewage treatment, which is suitable for its commercial generation and promotion [133,134,135,136].

### 5.2. Recovery and Challenges of Up-Conversion Fluorescent Probes

Up-conversion fluorescent probes have been fully used in many fields, but there are still some key problems to be solved when applying UCNPs. The sewage treatment detection needs to face a complex and variable detection environment. Therefore, the challenges of up-conversion fluorescent probes include selectively detecting target substances, improving the luminescence rate, and recycling. In addition, there is a lack of functional modification methods for UCNPs, and methods should be enriched which can make detection more convenient.

**Figure 14:**
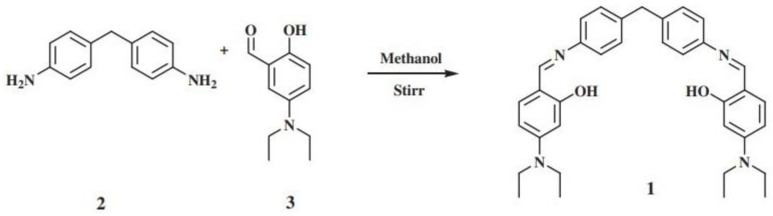
Structure diagram of multiple responsive fluorescent probes. Adapted with permission from K Kaur et al. [138]. 1: Zn^2+^ complex 2: NH_2_ ions 3: N ion and OH ion.

The recycling of fluorescent probes is the regeneration process of it. After the probe has selectively detected the target substance, using the principle of ionic competition between the intermediate product that was generated in situ and the specific ions to make the probe de-complexed to recover, it finally realizes the recycling of the probe [137]. Singh’s group [138] prepared a multiple response fluorescent probe, using Zn^2+^ to form a complex to detect metal atoms. Finally, PO_4_^3−^ was added to realize the regeneration of the fluorescent probe based on the principle of ionic competition, as shown in Figure 14. Tang’s group [139] also used a similar principle to add S^2−^ realizing the regeneration of fluorescent probes.

PFOA is an organic pollutant. In the existing research, HPLC–MS/MS is used to detect it. However, it has high cost and great damage to the environment. This work focuses on the application of UCNPs. On the one hand, UCNPs have the unique advantages of good photostability, adjustable emission wavelength, low toxicity, and good biocompatibility. The fluorescent probes prepared by UCNPs can effectively detect PFOA. On the other hand, UCNPs not only have low cost but also cause little secondary pollution to the environment. Therefore, this work has contributed to the detection of PFOA to a certain extent.

## 6. Conclusions and Outlook

The effective and simple detection of PFOA has importance to people’s lives. UCNPs are a good fluorescent material to detect PFOA, and NaYF_4_: Ho^3+^, Yb^3+^ is one of the better ones. Because NaYF_4_, as fluoride, is more suitable as the substrate of UCNPs to detect PFOA, and Ho^3+^ as the activator can better fit the characteristics of Yb^3+^. The up-conversion fluorescent probe prepared with UCNPs can effectively detect PFOA. The SiO_2_ modification and amino modification of the probe can make the probe dissolve in water faster and enhance luminous efficiency. Finally, the up-conversion fluorescent probe uses F-F interaction and electrostatic interaction to occur fluorescence quenching to detect PFOA.

The detecting method can be established in the laboratory, however, the preparation on a large scale is still a problem. In addition, it still has big obstacles to complete the experiments of recycling up-conversion fluorescent probes and selectively detecting PFOA. This method will have greatly positive influences on economics and the environment, because of the simplicity, ease, and efficiency of detecting PFOA in sewage and drinking water.

## Figures and Tables

**Table 1:** Comparison table of fluorescent probes.

Category	Advantages	Detection Limit	References
Small molecule fluorescent probes	Small molecule fluorescent probes have significant advantages such as high sensitivity, good membrane permeability, real-time in situ analysis, minimal biological damage, and easy handling.	1.05 × 10^−8^~5.8 × 10^−7^ mol/L	[40,41]
Polymeric fluorescent probes	It has a long fluorescence lifetime, good biocompatibility, and high quantum yield. It can also enhance intramolecular electron transport and increase the sensitivity of the probe.	0.17~2.3 μg/mL	[42,43]
Fluorescent probes based on nanomaterials	Compared with traditional fluorescent dyes, fluorescent nanomaterials not only have high fluorescence intensity and good light stability, but also have the characteristics of small size effect, quantum effect, and surface effect that are unique to nanomaterials, which can make up for the shortcomings of traditional fluorescent dyes.	6.4 × 10^−10^~1.225 × 10^−8^ mol/L	[44,45]

**Table 2:** Advantages and disadvantages of detection methods.

Category	Advantages	Detection Limit	References
Electrochemical Sensors	Electrochemical sensors can be used to detect chemical and biological compounds. Molecularly imprinted polymers (MIPs) functionalized sensors are effective in detecting PFASs, but may have the disadvantage of long detection times and high costs, as well as the inability to differentiate between similar molecules.	0.07~1.0 µg/L	[47,48,49]
Fluorescence Sensors	Fluorescent sensors occurred fluorescence quenching because of electron transfer between fluorescent dyes and PFASs, which can easily be detected using fluorescence spectrophotometers. However, this method can increase costs and cause environmental hazards	2.5 ppt~120 ppb	[50,51]
Optical Sensors/ Colorimetric sensors	Optical sensors/ Colorimetric sensors usually use organic dyes and can be detected by the naked eye based on visible color changes. Although it is not overly dependent on fluorescence spectrophotometry, co-existing atoms can interfere with the sensor. In addition, they require a good platform to improve sensor availability.	1 ppb~5 ppt	[52,53]
HPLC–MS/MS	HPLC–MS/MS is based on the principle of using liquid chromatography monopole mass spectrometry or multistage mass spectrometry as the separation and detection system. It has the advantages of low detection limits, high resistance to matrix effect interference, convenience, and rapid detection. The detection limit is approximately 0.07~0.15 μg/kg.	The method is expensive, costly, and highly specialized and requires a high level of operator expertise	[54,55,56,57]
HPLC–Q-TOF-MS	HPLC–Q-TOF-MS has a certain optimization in the ability to analyze samples when in a complex environment, which increase the correctness of detection. The detection limit is approximately 20~30 ng/L.	The method is too costly to be suitable for universal detection.	[28]
GC–MS	GC–MS is a tandem gas chromatograph and mass spectrometer method that offers the advantages of simplicity and convenience. The detection limit is approximately 15~40 ng/L.	PFOA is less volatile and needs to be derivatized before it can be detected by injection. The application of GC–MS is narrow, and the experimental process is accompanied by toxic substances.	[58,59,60]
Pre-column Derivatization GC	It is a method that improves on the shortcomings of GC–MS. The derivatization technique reduces the temperature of the target, and improves the signal required to detect the target. The detection limit is approximately 0.01~0.05 mg/kg.	The formation of by-products may cause greater difficulties in chromatographic separation. Impurities or interfering peaks can easily be introduced or samples lost during derivatization.	[34]
Rare earth up-conversion fluorescent probe	Fluorescence burst reaction of the probe with the detected substance, offering the advantage of simple, efficient, and highly sensitive detection. The detection limit is approximately 0.01~1.2 nmol/L.	PFOA is a perfluorinated compound. The method is not selectively detectable for PFOA	[61]

**Table 3:** Fluorescence quantum yields of fluorescent probes.

Fluorescent Probe	Quantum Yields	Reference
A highly selective Al^3+^ fluorescent probe	5.7% (366 nm)	[91,92]
Near-infrared H_2_S fluorescent probes	1.2% (530 nm)	[93]
Enhanced fluorescent probes for BSA	1.5% (645 nm)	[94]
Visualization of iron ion fluorescent probes	2.1% (610 nm)	[95]
Rare-earth up-conversion fluorescent probes	4.7% (980 nm)	[96]

**Table 4:** Advantages and disadvantages of UCNPs preparation methods. Adapted with permission from Qin YK et al. [115].

Method	Advantages	Disadvantages
Thermal decomposition method	The product has high quality, is monodisperse, and has excellent morphology	Reaction requires high temperature, no oxygen, and no water, easy to produce toxic gases
Coprecipitation method	Simple process, low cost, relative environmental protection, high yield	The reaction requires high-temperature calcination, and the particle size of the product is not uniform
Hydrothermal method	The synthesis method is efficient, simple, and morphologically controllable	The size of the product is commonly large
Sol–gel method	The product has good dispersion and uniform morphology	The product agglomerates easily
Microemulsion method	Particle size controllable, good morphology	The operation is complicated, the cost is high and the reaction condition is harsh

## Data Availability

No data were created or analyzed in this study. Data sharing is not applicable to this article.
